# Using linked administrative data to move from an individual unit to family units: An example from child protection policy to better inform family focused interventions.

**DOI:** 10.23889/ijpds.v7i3.1964

**Published:** 2022-08-25

**Authors:** Alicia Montgomerie, Rhiannon Pilkington, John Lynch

**Affiliations:** 1 University of Adelaide

## Objectives

To describe turning an incident based child protection system contacts and data (e.g. number of notifications) into child specific experiences and then into patterns of family experiences with child protection (CP) to inform family focused interventions and policy.

## Approach

Data was drawn from Better Evidence Better Outcomes Linked Data (BEBOLD), a whole-of-population linked de-identified administrative data platform for all children in South Australia born from 1991-2016 (n~500,000) as well as their families and carers. Data linked included birth registrations, perinatal statistics, child protection services, family health services, education, justice, hospitalisation, and welfare payments. Families have been defined in BEBOLD based on mother-child relationships identified via birth registrations. The mother is the index family member, and all children registered with the same mother are grouped into one family. We analysed family experiences of the child protection system over ten years from 2006 to the end of 2016 and described their sociodemographic characteristics.

## Results

Almost 1 in 4 families had contact with CP 10 years after the birth of their first child. Two-thirds of families in contact with CP had one or two children, but larger families are more likely to have contact (57.1% of families with five or more children compared to 21.9% for families with one child). Of the families that had CP contact, 56.5% had contact when they only had one child, 32.9% first had contact after birth of their second child, and 9% had contact had birth of third child. Families with only one child at first CP contact had a generally higher risk profile across a range of characteristics at birth than families who first had contact with two, or three or more children.

## Conclusion

The process requires a systematic change in the upstream data source, and we requested participating agencies to send us data in an agreed format. The receipt of files in standard format is pivotal for reducing the overall timeframes of HSDS creation and leverage it for policy and investment purpose.

